# Evaluation of the Effect of High-Speed Sintering and Specimen Thickness on the Properties of 5 mol% Yttria-Stabilized Dental Zirconia Sintered Bodies

**DOI:** 10.3390/ma15165685

**Published:** 2022-08-18

**Authors:** Kazumichi Nonaka, Mitsuji Teramae, Giuseppe Pezzotti

**Affiliations:** 1 Department of Research and Development, SHOFU INC, Higashiyama-ku, Kyoto 605-0983, Japan; 2 Ceramic Physics Laboratory, Kyoto Institute of Technology, Matsugasaki, Sakyo-ku, Kyoto 606-8585, Japan

**Keywords:** zirconia, dental material, high-speed sintering

## Abstract

High-speed sintering of zirconia has become essential to single-visit dental prosthetic treatments. This important prosthetic dentistry technique demands a translucent material tougher than porcelain. Previous studies on high-speed sintered zirconia did not take heat and material thickness into consideration. We evaluated pre-sintered specimen thickness and the effect of high-speed sintering on the properties of 5 mol% Y_2_O_3_-stabilized zirconia (5Y zirconia). High-speed sintered bodies of 5Y zirconia were evaluated by density measurements, translucency measurements, three-point flexural and fracture toughness tests, X-ray diffraction (XRD), and scanning electron microscopy (SEM). High-speed sintering reduced the translucency and mechanical properties of 5Y zirconia. XRD and SEM observation results clarified that these reductions were due to the change in crystal phase composition and to the increase in residual pores, respectively, both resulting from high-speed sintering. Moreover, in high-speed sintering, as the thickness of the specimen increased, the number and size of internal pores increased, and the translucency and strength decreased. The threshold value for avoiding a reduction in translucency and mechanical properties was found to lie at ~4.4 mm. From the above results, it was concluded that 5Y zirconia is not suitable for high-speed sintering applications.

## 1. Introduction

Dental prostheses are commonly produced from zirconia due to its high bending strength, fracture toughness, chemical stability, radiopacity, and biocompatibility [1,2]. The excellent mechanical properties of the zirconia sintered body are due to the toughening mechanism associated with stress-induced phase transformation from the metastable tetragonal phase to the stable monoclinic phase [3]. Therefore, in order to obtain excellent mechanical properties, the tetragonal phase must be retained at room temperature and stabilizers (i.e., CaO, MgO, CeO_2_, and Y_2_O_3_) must be added to the raw zirconia powder. Of these stabilizers, Y_2_O_3_ is used in most dental zirconia. The stabilizer concentration greatly influences the properties of zirconia. In general, the higher the concentration of the stabilizer, the higher the translucency, and conversely, the lower the mechanical properties [4,5]. However, Kulyk et al. [6,7] reported that mechanical properties showed ambiguous changes with respect to yttrium concentration. This difference is likely due to differences in the method of adding yttria to zirconia. In general, yttria is added by dissolving the yttrium source in a precursor solution of zirconia, whereas in [6,7], yttrium was added as a powder. The powder method results in yttrium non-uniformity and could be the cause of ambiguous changes in mechanical properties. These reports suggest that not only the yttrium concentration of the entire sintered body but also the local yttrium concentration and the accompanying crystal phase composition and microstructure affect the mechanical properties.

In the field of prosthodontics, 3 mol% Y_2_O_3_-stabilized zirconia (3Y zirconia), 4 mol% Y_2_O_3_-stabilized zirconia (4Y zirconia), and 5 mol% Y_2_O_3_-stabilized zirconia (5Y zirconia), are used most commonly. Selection is dependent on the intended use. For example, 5Y zirconia, which has high translucency, is used where aesthetic demands are high, such as in anterior teeth, while 3Y zirconia, which has excellent mechanical properties, is used where heavy loads are applied such as in long span bridges [8,9].

Zirconia dental prostheses are fabricated by employing computer-aided design and computer-aided manufacturing (CAD/CAM) systems. Since zirconia sintered bodies are difficult to process, they are generally produced after processing semi-sintered zirconia into the desired prosthesis shape and then sintering it for 6 to 10 h [10]. Since this long sintering cycle reduces the production efficiency of zirconia prostheses, efforts have been made to shorten the sintering time.

In recent years, several manufacturers have released furnaces for high-speed sintering. CEREC Speedfire (Dentsply Sirona) is a well-known example. This sintering furnace can densify zirconia with 30 min of microwave heating; so, it enables the completion of a zirconia prosthetic treatment within a single dental visit (One Visit Treatment) [11,12]. As mentioned above, more highly aesthetic prostheses require a zirconia material with high translucency (e.g., 5Y zirconia). This is because porcelain is usually not built onto dental prostheses made of monolithic zirconia, which are often used due to time and technical constraints. Generally, zirconia prostheses are made by dental technicians. In cases where aesthetic appearance is an issue, they build various shades of porcelain on zirconia-sintered bodies to give the appearance of natural teeth. The process of building porcelain shades requires long hours of work and a high level of technical skill. On the other hand, in a One Visit Treatment, the zirconia prosthesis must be made by the dentist in a short time. Therefore, in many cases, no porcelain is built on the zirconia sintered body.

It is well known that sintering schedules such as heating rate and holding time affect the translucency, relative density, particle size, and mechanical properties of sintered ceramics. Therefore, it is important to investigate the effect of high-speed sintering on the properties of dental zirconia. In fact, there are several studies on high-speed sintering effects on the properties of zirconia [13,14,15,16].

Jansen et al. [13] investigated the effect of high-speed sintering on 3Y zirconia and 4Y zirconia and reported that fast sintering reduces the translucency of 3Y zirconia and 4Y zirconia with low alumina content. Cokic et al. [14] investigated the effects of high-speed sintering on 3Y zirconia and 5Y zirconia, and reported that high-speed sintering reduces the mechanical properties of both and the translucency of 3Y zirconia. Lawson et al. [15] sintered three types of commercially available 5Y zirconia and reported that the effect of high-speed sintering on them depends on the type of 5Y zirconia. Li et al. [16] reported that high-speed sintered 3Y zirconia showed clinically suitable properties even in high-speed sintering.

However, in these studies, the effect of high-speed sintering could not be accurately evaluated because the sintering temperature of conventional and high-speed sintering often differs. Furthermore, the effect of high-speed sintering on the crystal phase of zirconia and its effect on the properties of zirconia were not fully discussed. In addition, there is a lack of knowledge of the reliability of thick dental prostheses because the effect on the properties of zirconia of the test piece thickness during sintering has not been discussed.

The aim of this study is to evaluate the effects of CEREC Speedfire high-speed sintering on the translucency and mechanical properties of 5Y zirconia at various specimen pre-sintered thicknesses, and to clarify the causes from crystal structure and microstructure assessments.

## 2. Materials and Methods

### 2.1. Preparation of Test Piece

**Figure 1:**
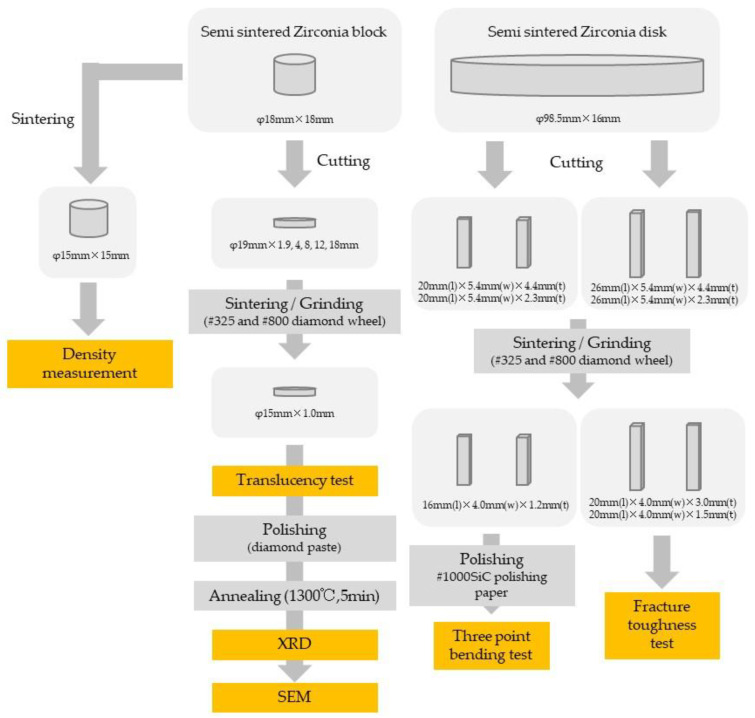
Specimen preparation flowchart and sample sizes (the mark “φ” denotes “diameter”).

Specimen dimensions and fabrication procedures are summarized in Figure 1, Table 1 and Table 2. Specimens of several thicknesses were prepared to investigate the effect of specimen pre-sintered thickness (cf. labels in inset to Figure 1).

#### 2.1.1. Preparation of Pre-Sintered Test Piece

Zpex Smile (Tosoh, Tokyo, Japan) were used as raw materials for 5Y zirconia containing 0.05 wt% of alumina. The raw material was press-molded by a uniaxial press molding machine (Sansho Industry, OSAKA, JAPAN) to obtain a block having a diameter of 19 mm × 19 mm and a disc having a diameter of 100 mm × 17 mm. After that, CIP treatment was performed at 200 MPa using a cold hydrostatic isotropic press (Sansho Industry, Osaka, Japan).

The obtained zirconia block/disc was degreased at 500 °C in an inert gas oven (INH-21CD, KOYO THERMO, Nara, Japan) and then fired in a firing furnace (SC-3035F, MOTOYAMA, Osaka, Japan) at 970 °C, to obtain a semi-sintered zirconia block/disk (φ18 mm × 18 mm/φ98.5 mm × 16 mm).

The semi-sintered zirconia block/disk was cut with a cutting machine (Secotom, Struers, Ballerup, Denmark), and the surface was polished with SiC water-resistant polishing paper (# 1000), to reach a shape suitable for each test.

#### 2.1.2. Sintering of Test Piece

**Figure 2:**
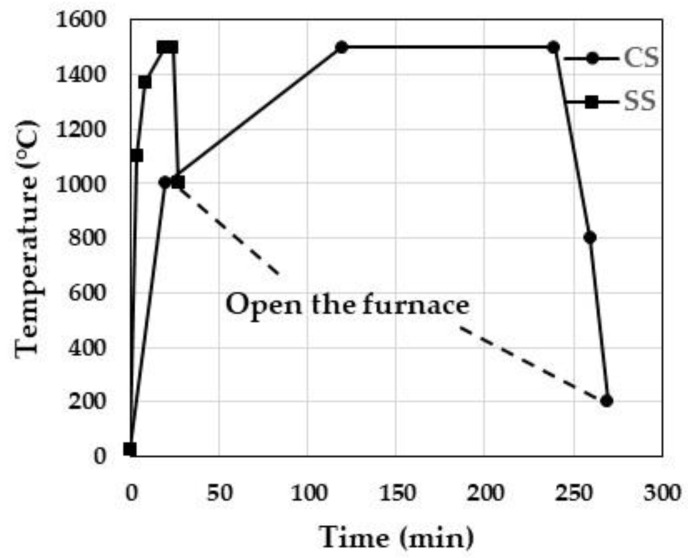
Sintering schedules of conventional sintering (CS) and speed sintering (SS).

Each test piece was subjected to high-speed sintering (SS: 1500 °C, 5 min) or conventional sintering (CS: 1500 °C, 2 h). CEREC Speed fire (Dentsply Sirona, Charlotte, NC, USA) was used for high-speed sintering, and AUSTROMAT 674i (DEKEMA, Freilassing, Germany) was used for conventional sintering. Each sintering process was performed according to the schedule shown in Figure 2.

#### 2.1.3. Processing of Sintered Body

Each sintered body was ground by a surface grinding machine (GRIND-X PFG500II, OKAMOTO, Gunma, Japan) using diamond wheels (# 325, # 800) and processed to the dimensions corresponding to each test (Figure 1 and Table 2). Specimens for XRD and SEM were polished with dental zirconia polishing paste Zirco Shine (SHOFU INC., Kyoto, Japan), and for the three-point flexural test, they were polished with water-resistant SiC polishing paper (# 1000). Specimens for XRD and SEM analyses were then heat-treated at 1300 °C for 5 min, to remove the effects of phase transformations and residual stresses due to mechanical polishing.

### 2.2. Density Measurement

The density of the specimens was measured by the Archimedes method (n = 3). The measurement was carried out in accordance with JIS R 1634: 1998. From the obtained density, the relative density was calculated by the following equation:

(1) $$\begin{array}{c} \mathrm{Relative}\;\mathrm{density}=\frac{\rho }{\rho_{0}}\times 100,\\ \rho_{0}=\frac{100}{\left(\frac{A}{\rho_{A}}+\frac{100-A}{\rho_{x}}\right)}, \end{array}$$

*A*: content of alumina (wt%);
*Ρ*: measured density (g/cm^3^);
*ρ*_0_: theoretical density (g/cm^3^);
*ρ_A_*: theoretical density of alumina;
*ρ_x_*: theoretical density of zirconia containing 5.5 mol% of yttria (6.0254 g/cm^3^ [17]).

### 2.3. Crystallographic Characterization

Crystal structure characterization of the specimens was performed by X-ray Diffraction (Multi Flex, Rigaku, Tokyo, Japan). The measurement was performed using CuKα as the X-ray source, tube current and tube voltage of 40 mA and 40 kV, respectively, 2θ range of 20–120°, scan step of 0.02°. A Rietveld analysis was performed for each diffraction profile using Rietan-FP [18]. For the initial structural model of Rietveld analysis, two types of tetragonal phases with different yttrium contents were used as described in a report [19] by Belli et al. (Table 3).

The tetragonality *c/a* was calculated from the lattice constant refined by Rietveld analysis. The yttria concentration in tetragonal zirconia was calculated using the following equations, as given by Miller et al. [20]:

(2) $${\mathrm{YO}}_{1.5}\;\left(\mathrm{mol}\%\right)=\frac{1.0223-c/a}{0.001319},$$

(3) $$Y_{2}O_{3}\;\left(\mathrm{mol}\%\right)=\frac{{\mathrm{YO}}_{1.5}\;\left(\mathrm{mol}\%\right)/100}{2-{\mathrm{YO}}_{1.5}\;\left(\mathrm{mol}\%\right)/100}\cdot 100.$$

**Table 3:** ICDD cards used for refinement of the specimen.

Phase	Chemical Formula	Space Group	ICDD Number	Authors
4Y low yttrium tetragonal	(ZrO_2_)_0.96_(Y_2_O_3_)_0.04_	P4_2_/nmc	00-060-0503	Yamashita et al. [21]
5Y tetragonal	(ZrO_2_)_0.95_(Y_2_O_3_)_0.05_	P4_2_/nmc	01-070-4428	Lamas and Walsöe De Reca [22]

### 2.4. Microscopic Characterization

Microscopic characterization was performed by SEM (JSM6390LA, JEOL, Tokyo, Japan) at an acceleration voltage of 15 kV. The specimens used for XRD were the same as those used for SEM observation. The specimens were Au-coated to prevent charge-up.

### 2.5. Translucency

The translucency was evaluated by computing the *TP* (Translucency Parameter) according to the following equation:

(4) $$\mathrm{TP}={\left[({L^{*}}_{w}-{L^{*}}_{b})+({a^{*}}_{w}-{a^{*}}_{b})+({b^{*}}_{w}-{b^{*}}_{b})\right]}^{1/2},$$

L*, a*, b*: CIE L* a* b* value;
Subscript *w*: measured with the white background;
Subscript *b*: measured with the black background.

The L* a* b* of each specimen was measured by a spectroscopic colorimeter (CM-5, Konica Minolta, Hannover, Germany). The specimens were measured on a black and white background (n = 2).

### 2.6. Three-Point Flexural Test

The three-point flexural test was conducted in accordance with ISO6872 (Dentistry-Ceramic materials) (n = 10). In order to investigate the effect of the specimen pre-sintered thickness on the strength, specimens made from two types of semi-sintered bodies with different thicknesses were tested (Figure 1). The test was carried out using a universal testing machine (Instron 5967, INSTRON, Norwood, MA, USA) under the conditions of a span of 12 mm and a crosshead speed of 1.0 mm/min. Three-point flexural strength *σ* (MPa) was calculated according to the following equation in accordance with ISO 6872:

(5) $$\sigma =\frac{3\mathrm{PL}}{{2\mathrm{wb}}^{2}},$$

*P*: breaking load (N);
*L*: test span (mm);
*w*: width of the specimen (mm);
*b*: thickness of the specimen (mm).

### 2.7. Fracture Toughness Test

The fracture toughness test used the SEVNB method in accordance with ISO 6872 (n = 5). In this test, two types of specimens with different thicknesses (22 mm (l) × 4.0 mm (w) × 3.0 mm (b) and 22 mm (l) × 4.0 mm (w) × 1.5 mm (b)) were used in order to investigate the effect of the specimen pre-sintered thickness on the fracture toughness. The V-notch was applied along the thickness direction of each test piece to a depth of 0.8 to 1.2 mm, an angle of less than 20°, and a tip diameter of less than 20 μm using a razor blade (FH-10B, FEATHER, Osaka, Japan). The test was carried out using a universal testing machine (Instron5967, INSTRON, Norwood, MA, USA) under the conditions of a span of 16 mm and a crosshead speed of 0.5 mm/min. The fracture toughness value, *K*_1C_ ($\mathrm{MPa}\sqrt{m}$), was calculated according to the following equation, in accordance with ISO 6872 [23]:

(6) $$\begin{array}{c} K_{1C}=\frac{F}{b\sqrt{w}}\times \frac{S}{w}\times \frac{3\sqrt{a}}{{2(1-\alpha )}^{1.5}}Y,\\ Y=\frac{1.99-a(1-a)(2.15-3.93a+2.7a^{2})}{\left(1+2a\right)}, \end{array}$$

*F*: fracture load (MN);
*S*: test span (mm);
*w*: specimen thickness (mm);
*b*: specimen width (mm);
*α*: relative V-notch depth (mm).

$$\alpha =\frac{a}{w}$$

*a*: average notch depth (mm);

$$a=\frac{a1+a2+a3}{3}$$

### 2.8. Statistical Analysis

The *t*-test was used to compare 5Y-SS and 5Y-CS in relative density and TP (specimen pre-sintered thickness 1.9 mm). Flexural strength and fracture toughness were analyzed using two-way analysis of variance (two-way ANOVA). The level of significance was set at 0.05 for all analyses.

## 3. Results and Discussion

### 3.1. Density

**Figure 3:**
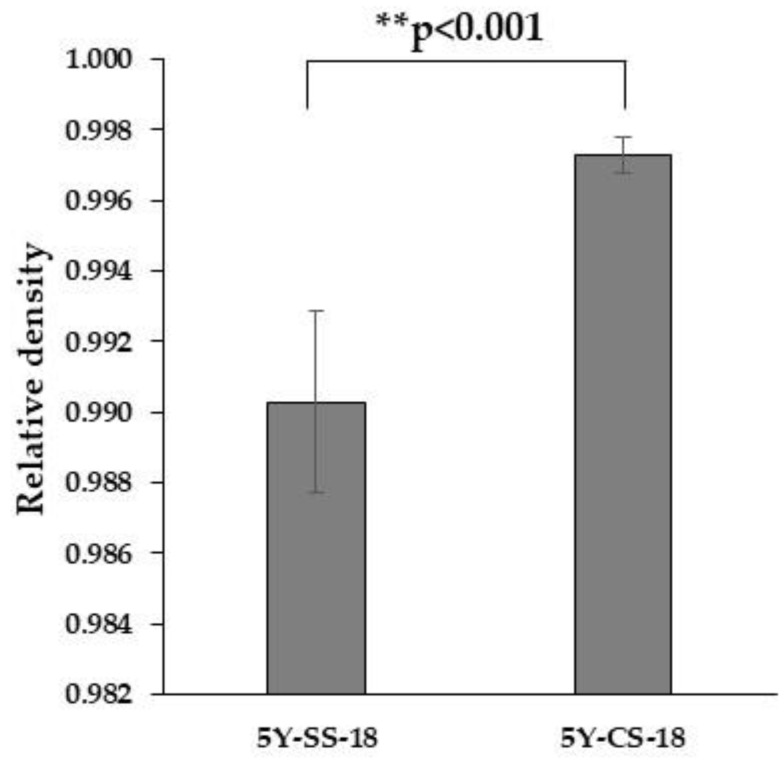
Relative densities of 5Y- SS and 5Y-CS. Two asterisks refer to statistical significance.

Figure 3 shows the relative densities of 5Y-SS-18 and 5Y-CS-18. The relative density of 5Y-SS was significantly lower than that of 5Y-CS. This result indicates that more pores remain in 5Y-SS than in 5Y-CS, suggesting that in 5Y zirconia, high-speed sintering results in inadequate densification.

Contrary to this study, Cokic et al. [14] reported that fast sintering did not affect the density of 5Y zirconia. The cause of this difference is considered to be the difference in sample size and the difference in the type of 5Y zirconia. In this study, thick specimens of φ15 mm × 15 mm (φ18 × 18 mm before sintering) were used for density measurement, whereas the aforementioned researchers used thin specimens, 12 mm × 12 mm × 0.5 mm (15 mm × 15 mm × 3.5 mm before sintering). High-speed sintered 5Y zirconia increases residual pores as the specimen pre-sintered thickness increases (see Section 3.3). Therefore, the difference between this study and the study by Cokic et al. could be due to the difference in specimen thickness. In addition, while Zpex Smile was used as 5Y zirconia in this study, they used Katana STML (Kuraray Noritake, Tokyo, Japan). These types of zirconia semi-sintered bodies differ in zirconia primary particle size, the type and concentration of additives (i.e., alumina), and the uniformity of yttrium, which in turn affect the sintering behavior.

### 3.2. Crystallographic Characterization

**Figure 4:**
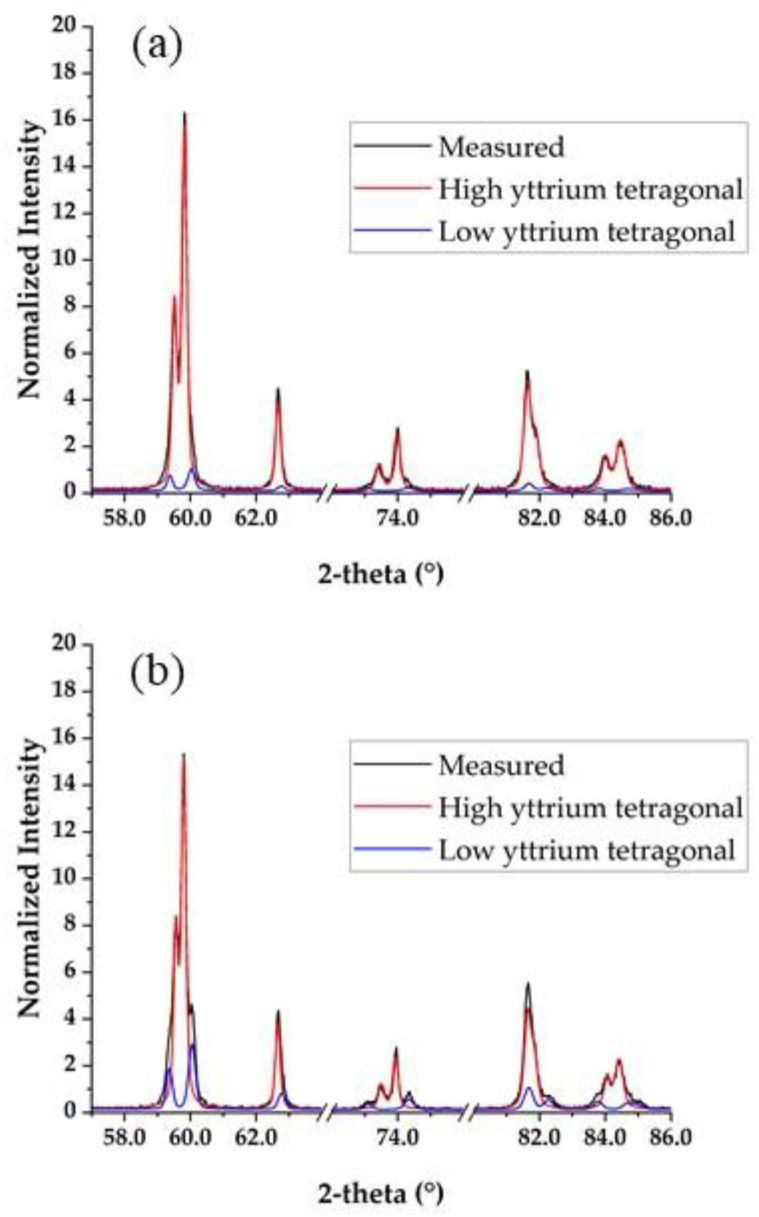
X-ray diffraction patterns of: (**a**) 5Y-SS-1.9, (**b**) 5Y-CS-1.9.

Figure 4 shows the XRD patterns of 5Y-SS-1.9 and 5Y-CS-1.9. Both 5Y-SS and 5Y-CS could be identified as the tetragonal phases, high yttrium tetragonal and low yttrium tetragonal. Table 4 shows the weight ratios of the two tetragonal phases and their yttrium concentrations. The crystal phase composition of 5Y-CS is in good agreement with the values previously reported by Belli et al. [19]. In 5Y-SS, however, the difference in yttria concentration of the two tetragonal phases was smaller and their content difference was larger than that of 5Y-CS. This is considered to be due to the high heating rate and the short holding time. Matsui et al. [24] reported that in the sintering of 3Y zirconia, yttrium segregates at the grain boundaries at 1300 °C or higher and two regions with yttrium concentration of 6 to 7 mol% and ~2 mol% are formed at 1500 °C. Similar phase separation has been confirmed in 4Y zirconia [21]. Considering that yttrium segregation and phase separation occur in 5Y zirconia by the same mechanism, in 5Y-SS, yttrium segregation and phase separation are suppressed because of the high heating rate and short holding time (5 min). As a result, it is probable that the crystal phase composition of 5Y-SS differed from that of 5Y-CS.

As previously mentioned, Cokic et al. reported results that differed from those of the present study regarding the effect of high-speed sintering on the crystal phase composition of 5Y zirconia [14]. In their study, high-speed sintering did not affect the crystalline phase composition of 5Y zirconia. Again, this could be due to the different types of 5Y zirconia semi-sintered bodies used (cf. Section 3.1). In high-speed sintering, the high-temperature interval of time, in which the diffusion of ions is promoted, is short, and the state of the material before sintering could greatly affect post-sintering characteristics.

**Figure 5:**
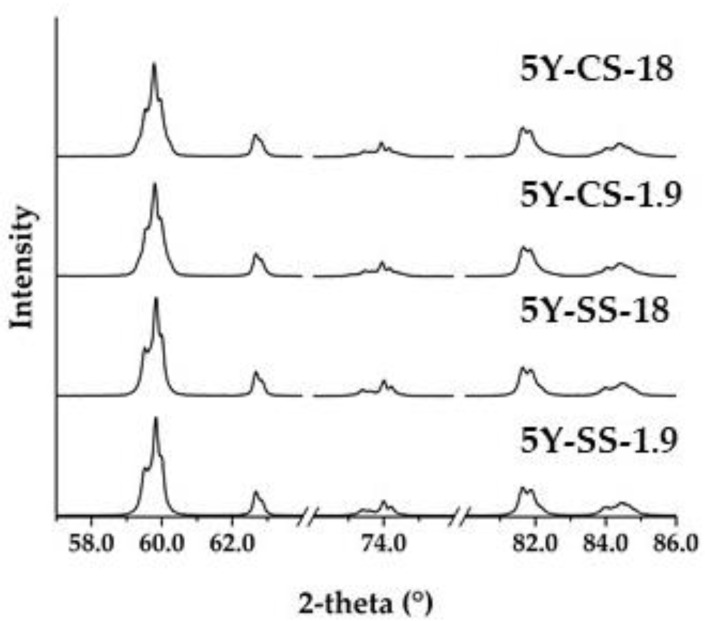
X-ray diffraction patterns of 5Y-SS-1.9, 18 and 5Y-CS-1.9, 18.

Figure 5 shows the X-ray diffraction patterns of the specimens with thicknesses of 1.9 mm and 18 mm before sintering. In both 5Y-SS and 5Y-CS, no change was observed in the X-ray diffraction pattern depending on the specimen pre-sintered thickness. This indicates that the thickness of the specimen does not affect the crystal phase in any of the sintering methods.

### 3.3. Microscopic Characterization

**Figure 6:**
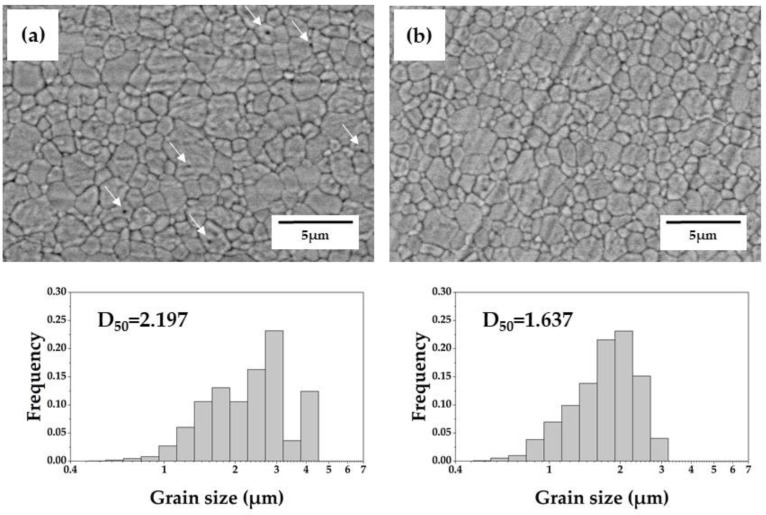
Scanning electron micrographs and grain size histograms of: (**a**) 5Y-SS-1.9, (**b**) 5Y-CS-1.9. Arrows in the micrograph indicate pores.

Figure 6 shows SEM images of 5Y-SS-1.9 and 5Y-CS-1.9. Almost no pores were confirmed in 5Y-CS, whereas some pores were confirmed in 5Y-SS (arrow in Figure 6a). Possibly, this is because the pores were not discharged and remained due to the rapid densification and grain growth associated with high-speed sintering. In addition, the median diameter of 5Y-SS grains was larger than that of 5Y-CS grains. In particular, the number of particles 4 μm or larger increased remarkably, suggesting non-uniform grain growth. It is known that the grain size of zirconia ceramics is affected by sintering conditions such as sintering temperature and holding time [6,7,25,26]. In many reports, the lower the sintering temperature and the shorter the holding time, the smaller the grain size [27,28,29]. Similar to the results of this study, Cokic et al. reported that the grain size of 5Y zirconia increased by high-speed sintering [14]. The cause of these differences in grain growth behavior is not clear, but since only Cokic et al. used the same sintering furnace (Speedfire) as in this study, it is possible that the grain-coarsening observed in 5Y-SS is a phenomenon specific to Speedfire.

**Figure 7:**
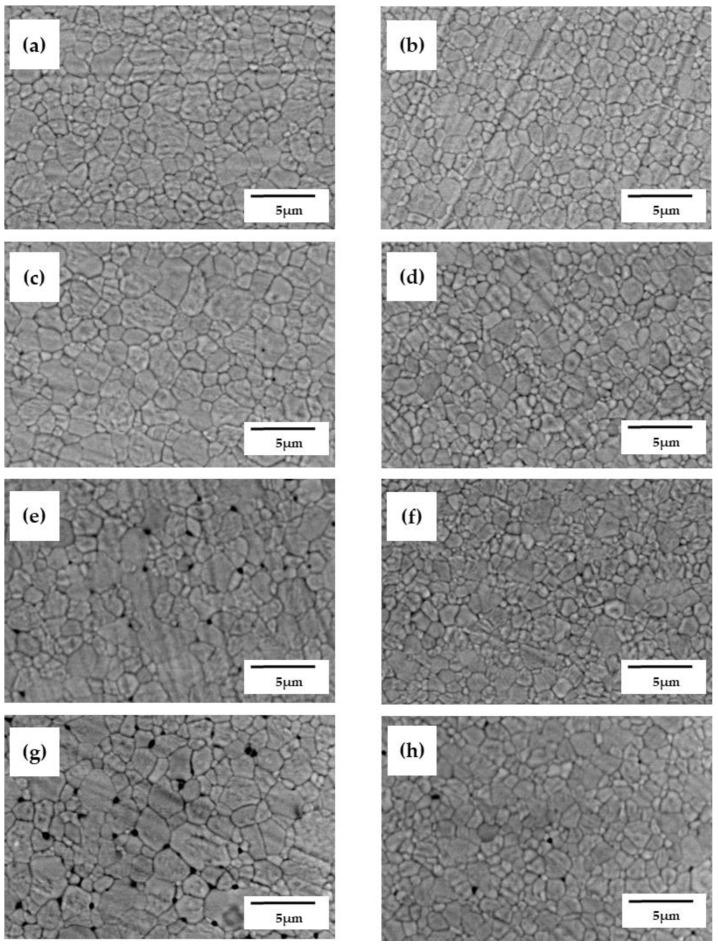
Scanning electron micrographs of: (**a**,**c**,**e**,**g**) 5Y-SS-1.9, 4.0, 8.0, 18, (**b**,**d**,**f**,**h**) 5Y-CS-1.9, 4.0, 8.0, 18.

**Figure 8:**
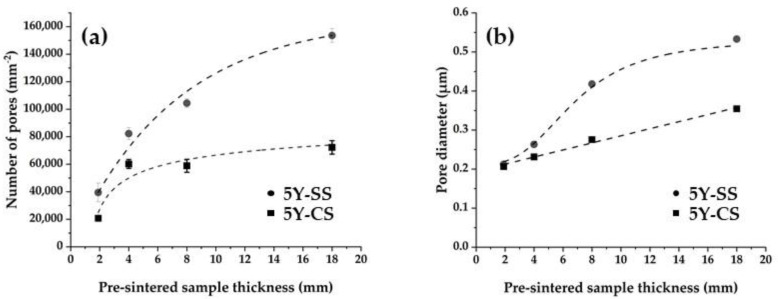
Relationship between the specimen’s pre-sintered thickness and (**a**) the number and (**b**) the diameter of pores.

Figure 7 shows the SEM images of 5Y-SS and 5Y-CS with a specimen thickness of 1.9 mm, 4.0 mm, 8.0 mm, and 18 mm before sintering. Figure 8 shows the relationship between the specimen pre-sintered thickness and the number and diameter of pores. Counting the number of pores in the SEM image in each of the three views of each specimen, calculating the average, and converting it per unit area was the method used for pore number calculations. In both 5Y-SS and 5Y-CS, the pores increased as the thickness of the pre-sintered specimen increased, especially in 5Y-SS. This indicates that the core region of the thick specimen is not sufficiently densified, especially in 5Y-SS. In addition, an increase in pore size was observed in 5Y-SS-8.0, 5Y-SS-18, and 5Y-CS-18.

**Figure 9:**
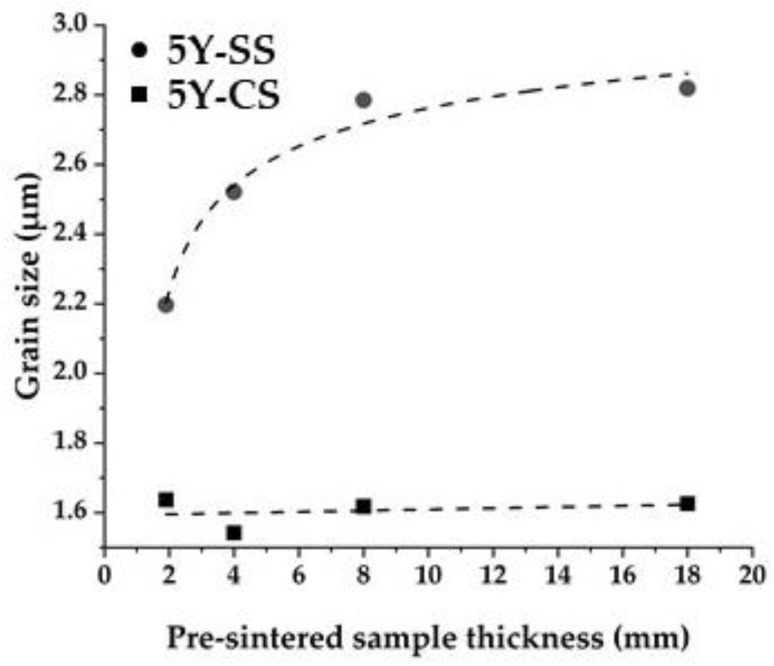
Relationship between the specimen’s pre-sintered thickness and the crystal grain size.

Figure 9 shows the relationship between the specimen’s pre-sintered thickness and the crystal grain size. In 5Y-CS, the grain size did not correspond to the thickness, whereas in 5Y-SS, the grain size increased as the specimen thickness increased. This suggests that in 5Y-SS, ion diffusion is promoted inside the specimen rather than near the surface. This could be because the temperature inside 5Y-SS is higher than that of the surface of the specimen. Since the Speedfire used for sintering 5Y-SS is a microwave heating furnace, the specimen itself generates heat during sintering, and there is no heat source around the test piece. Therefore, the temperature inside the test piece is inevitably higher than that near the surface, known as the “inverse temperature gradient” [30]. As a result, it is considered that the central part of the specimen was rapidly densified and grain-grown as compared with the surface, and a large number of pores were generated in that area. Charmond et al. also reported non-uniform grain growth in microwave sintering [31].

Since only a small amount of alumina was added to the zirconia raw material used in this study, precipitation of alumina particles was not observed in any specimen. The solubility of alumina in 8 mol% yttria-stabilized zirconia is reported to be ~1 wt% at 1500 °C [32]. Although 8Y zirconia and 5Y zirconia may have different alumina solubility limits, it is considered that alumina is completely dissolved at the grain boundary of zirconia, because the concentration of alumina in this study is about 0.05 wt%, which is much lower than the solubility limit. It has been reported that, in polycrystalline zirconia, the dissolved alumina is mainly segregated at grain boundaries [33]. Therefore, we shall consider alumina as segregated at the grain boundaries in all specimens in this study.

### 3.4. Translucency

**Figure 10:**
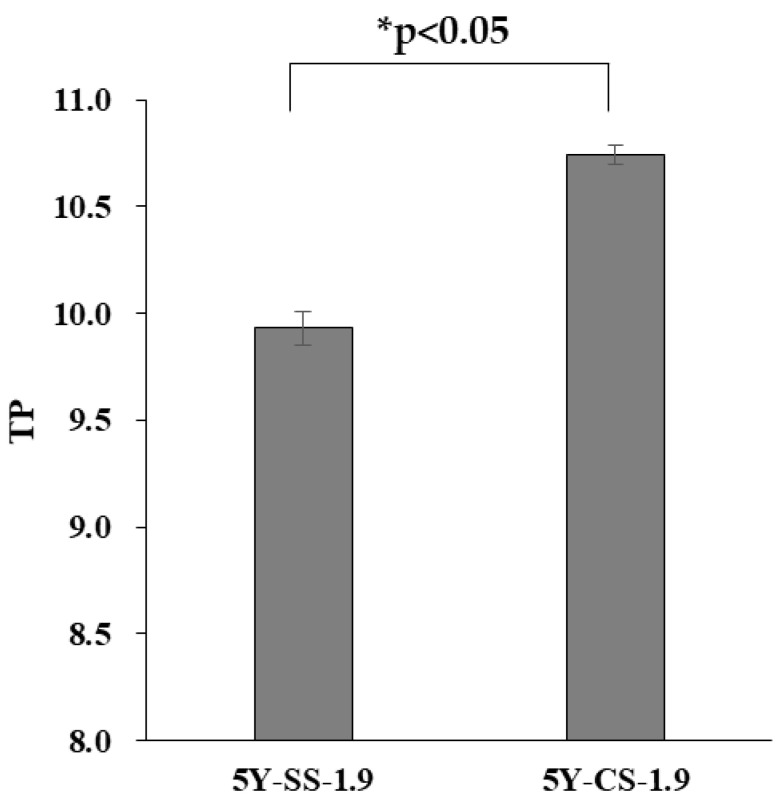
TP of 5Y-SS-1.9 and 5Y-CS-1.9. The asterisk refers to statistical significance.

Figure 10 shows the TP of 5Y-SS and 5Y-CS. The TP of 5Y-SS was significantly lower than that of 5Y-CS, suggesting that high-speed sintering reduces the translucency of 5Y zirconia. The residual pores can be considered the main cause of this decrease in translucency. Density measurements and SEM observations suggest that 5Y-SS has more residual pores than 5Y-CS. It is probable that these residual pores became a light-scattering source and reduced the translucency. The translucency of zirconia is strongly influenced by the size and concentration of pores due to zirconia’s high refractive index [34].

The crystal phase composition is considered a factor affecting the translucency. Ceramic polycrystals generally exhibit higher translucency as they contain more optically isotropic crystal phases. From the XRD results, both 5Y-SS and 5Y-CS consist of tetragonal phases that are optically anisotropic. In the tetragonal phase, the lower the tetragonality, the closer to the optically isotropic cubic phase the sample becomes. Therefore, the light scattering due to optical anisotropy is reduced.

Zhang et al. compared the translucency of a 5Y zirconia sintered body prepared by mixing 3Y zirconia powder and 8Y zirconia powder with the 5Y zirconia sintered body prepared from the uniform 5Y zirconia powder, and reported that the former was less translucent than the latter. Their study concluded that the higher the yttrium concentration of low yttrium tetragonal crystals, the higher the translucency when the overall yttrium concentration is the same [35].

However, this study showed the opposite result to that reported by Zhang et al.: 5Y-SS had lower translucency than 5Y-CS, despite the high yttrium concentration of the low-yttrium tetragonal phase. This could be because, under the conditions of this experiment, the difference in the number of residual pores had a greater effect on translucency than the crystallographic difference.

**Figure 11:**
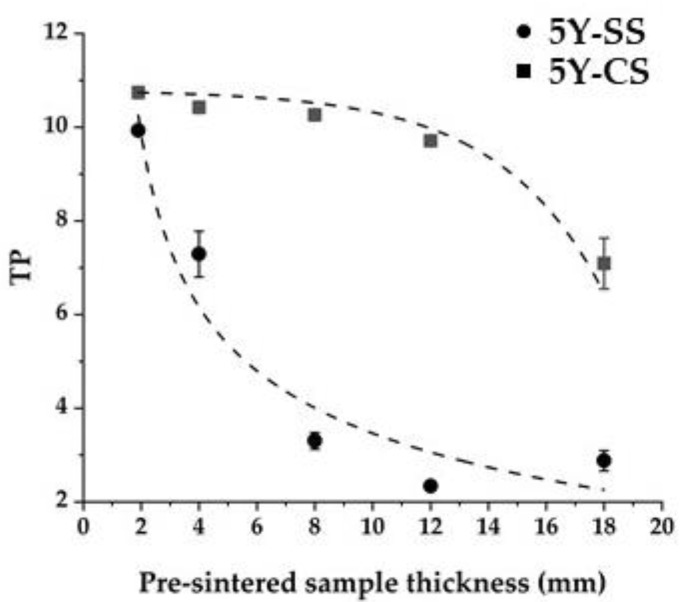
Relationship between the specimen’s pre-sintered thickness and TP.

Figure 11 shows the relationship between the thickness of the specimen before sintering and translucency. In both 5Y-SS and 5Y-CS, the translucency decreased as the thickness of the pre-sintered specimen increased. This decrease in translucency was mainly due to the increase in pores since the number of pores increased as the pre-sintered thickness of the specimen increased (Figure 8). In addition, the pore size in the 5Y-SS sample with a thickness of 8 mm or more, and in the 5Y-CS sample with a thickness of 18 mm, which both show a marked decrease in translucency, has increased. This suggests a relationship between pore size and translucency.

High translucency is the only reason to use 5Y zirconia, which is less strong than 3Y and 4Y zirconia, in dental prostheses. Therefore, it can be concluded that 5Y zirconia is not suitable for high-speed sintering applications.

### 3.5. Mechanical Properties

**Figure 12:**
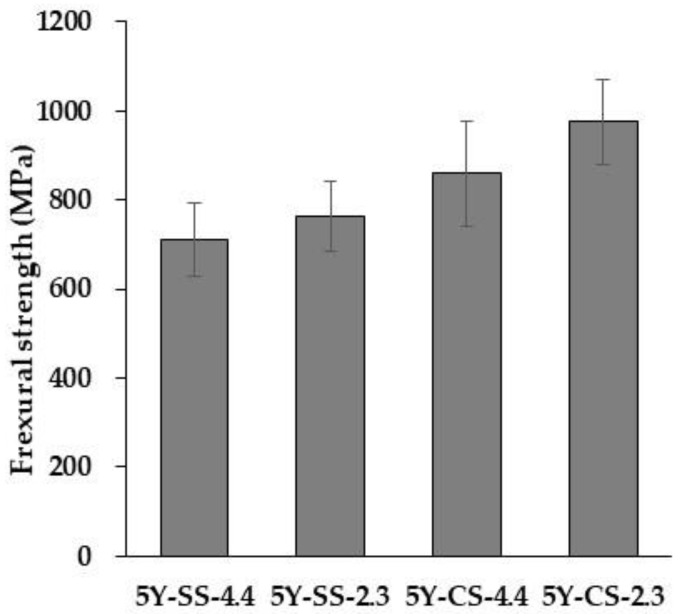
Tree-point flexural strengths of 5Y-SS and 5Y CS.

**Figure 13:**
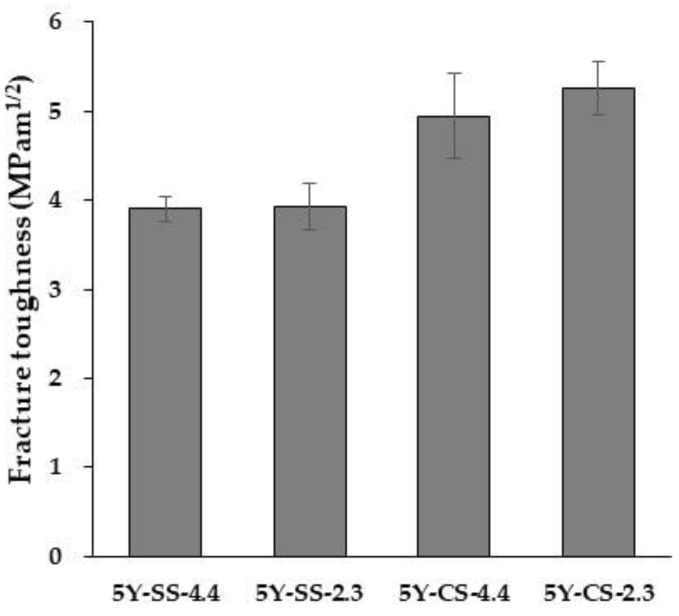
Fracture toughness of 5Y-SS and 5Y CS.

The results of the three-point flexural test and the fracture toughness test are shown in Figure 12 and Figure 13, and the results of each two-way ANOVA are shown in Table 5 and Table 6, respectively. In the flexural strength, the sintering method and specimen thickness showed a significant difference, while cross analyses including specimen thickness and sintering method (cf. Sintering method * Specimen thickness) did not differ significantly. On the other hand, as regards fracture toughness, only different sintering methods produced a significant difference.

Crystal phase composition and microstructure are considered to be factors leading to low mechanical properties in 5Y-SS. Firstly, 5Y-SS has a lower content of low yttrium tetragonal and a higher yttria concentration in it than 5Y-CS. Therefore, the contribution of toughening caused by the “tetragonal → monoclinic stress-induced phase transformation” is smaller than that of 5Y-CS [19]. As a result, flexural strength and fracture toughness decreased. Secondly, 5Y-SS has more residual pores than 5Y-CS (Figure 7 and Figure 8). Residual pores become fracture origins in polycrystalline ceramics. Therefore, flexural strength decreased in 5Y-SS.

As for flexural strength, the specimen thickness induced a significant difference: 5Y zirconia has a reduced flexural strength in thick specimens. This is because the residual pore concentration increases toward the center of the specimen. Moreover, since the fracture toughness does not change with specimen pre-sintered thickness, there is no difference in the degree of toughening caused by stress-induced phase transformation.

5Y-SS-4.4 showed a flexural strength of 712 MPa. This strength value complies with ISO 6872 Class 4 (>500 MPa; approved for use with 3-unit prostheses, those connecting 3 teeth). One Visit Treatment is not available for cases requiring prostheses larger than a 3-unit one, so 5Y-SS-4.4 seems to have a clinically suitable strength value. Even so, 5Y zirconia cannot be used for high-speed sintering because in some cases, e.g., pontic bridge, the dental prosthesis may be as thick as 10 mm. As mentioned above, the residual pores of 5Y-SS increase both in density and size as the specimen thickness increases (Figure 7 and Figure 8). Therefore, specimens with a pre-sintered thickness greater than 4.4 mm could have significantly reduced strength.

### 3.6. Effect of Chemical Composition

This study shows that the translucency and mechanical properties of 5Y zirconia are greatly affected by high-speed sintering. On the other hand, previous studies on high-speed sintering did not show a significant reduction in translucency and mechanical properties of 3Y zirconia and 4Y zirconia [13,14,29]. This suggests that the higher the yttria content, the greater the high-speed sintering effect. Matsui et al. [36] reported that as yttrium concentration increased, the sintering rate in the early stage of sintering decreased, and the sintering rate after the middle stage of sintering increased. According to this report, 5Y zirconia sinters more slowly in the early stage of sintering and more rapidly after the middle stage of sintering, compared to 3Y zirconia and 4Y zirconia. In the high-speed sintering of this study, the heat is extremely high compared to conventional sintering (12 times higher at 1100 °C to 1370 °C). Because 5Y zirconia rapidly densifies after the middle stage of sintering, at such a high-speed temperature increase, pores could not be sufficiently eliminated and many remained.

A similar discussion could be made for alumina. Matsui et al. reported that the addition of alumina to yttria-stabilized zirconia favors volume diffusion over grain boundary diffusion and increases the sintering rate [37]. The addition of alumina can adversely affect high-speed sintered zirconia as well as zirconia with high concentrations of yttria.

## 4. Conclusions

This study revealed that high-speed sintering with Speedfire reduces the translucency and mechanical properties of 5Y zirconia. Crystallographic characterization by XRD and microstructure observation by SEM showed that these decreases were due to changes in crystal phase composition due to suppression of yttrium segregation and increased residual pores.

Evaluation of pre-sintered specimens of various thickness revealed that the amount and size of residual pores inside 5Y zirconia increase as the specimen’s pre-sintered thickness increases, resulting in reduced translucency and mechanical properties. Especially as regards high-speed sintering, 5Y zirconia is not suitable for thicker dental prostheses because the translucency and strength are significantly reduced when the pre-sintered thickness is 8 mm or more.

Discussions on the relationship between chemical composition and the effects of high-speed sintering suggested that high yttrium concentrations and the addition of alumina could adversely affect high-speed sintered zirconia.

From the above results, we conclude that 5Y zirconia is not suitable for high-speed sintering applications.

## Figures and Tables

**Table 1:** Raw material powders and sintering methods of specimens. The number at the end of the specimen’s name indicates the thickness of the specimen before sintering.

Sample Name	Raw Material Powder	Chemical Composition (wt%) *	Sintering Method
5Y-SS-××	Zpex Smile	ZrO_2_: 88.84 Y_2_O_3_: 9.29 Al_2_O_3_: 0.049 HfO_2_: 1.82	High-Speed
5Y-CS-××	Zpex Smile		Conventional

* Analysis value of the manufacturer.

**Table 2:** Specimen dimensions in each test (the mark “φ” denotes “diameter”).

Measurement	Specimen Dimensions	
	Before Sintering	At The Time of Measurement
Density	φ18 mm × 18 mm	φ15 mm × 15 mm
XRD	φ18 mm × 1.9 mm and 18 mm	φ15 mm × 1.0 mm
SEM	φ18 mm × 1.9, 4.0, 8.0 and 18 mm	φ15 mm × 1.0 mm
Translucency	φ18 mm × 1.9, 4.0, 8.0, 12 and 18 mm	φ15 mm × 1.0 mm
Three-point flexural test	20 mm (l) × 5.4 mm (w) × 2.3 mm (t) 20 mm (l) × 5.4 mm (w) × 4.4 mm (t)	16 mm (l) × 4.0 mm (w) × 1.2 mm (t)
Fracture toughness test	26 mm (l) × 5.4 mm (w) × 4.4 mm (t) 26 mm (l) × 5.4 mm (w) × 2.3 mm (t)	20 mm (l) × 4.0 mm (w) × 3.0 mm (t) 20 mm (l) × 4.0 mm (w) × 1.5 mm (t)

**Table 4:** Tetragonality, Y_2_O_3_ contents (calculated by Equations (2) and (3)) and phase contents (Rietveld).

		5Y-SS	5Y-CS	Previous Report [19]
high yttrium tetragonal	tetragonality	1.0063	1.0050	-
	Y_2_O_3_ (mol%)	6.44	7.03	7.01–7.16
	phase content ratio	0.87	0.74	0.63–0.67
low yttrium tetragonal	tetragonality	1.0137	1.0145	-
	Y_2_O_3_ (mol%)	3.36	3.04	2.95–3.10
	phase content ratio	0.13	0.26	0.33–0.37

**Table 5:** Results of two-way ANOVA statistical analysis for three-point flexural test.

Source	SS	df	MS	F	*p*	*: *p* < 0.05 **: *p* < 0.01
Sintering method	319,942.53	1	319,942.53	35.18	<0.001	**
Specimen thickness	70,205.46	1	70,205.46	7.72	0.0086	**
Sintering method * Specimen thickness	10,055.90	1	10,055.90	1.11	0.3000	
Error	327,423.94	36	9095.11			
Total	727,627.83	39				

*Note.* SS: Sum of squares; df: degree of freedom; MS: Mean square; F: F value; *p*: *p* value.

**Table 6:** Results of two-way ANOVA statistical analysis for fracture toughness test.

Source	SS	df	MS	F	*p*	*: *p* < 0.05 **: *p* < 0.01
Sintering method	7.0615	1	7.0615	69.90	<0.001	**
Specimen thickness	0.1460	1	0.1460	1.44	0.2468	
Sintering method * Specimen thickness	0.1030	1	0.1030	1.02	0.3277	
Error	1.6165	16	0.1010			
Total	8.9269	19				

*Note*. SS: Sum of squares; df: degree of freedom; MS: Mean square; F: F value; *p*: *p* value.

## Data Availability

The datasets generated and/or analyzed during the current study are available from the corresponding author on reasonable request.
